# Decision-Making in Patients with Hyperthyroidism: A Neuropsychological Study

**DOI:** 10.1371/journal.pone.0129773

**Published:** 2015-06-19

**Authors:** Lili Yuan, Yanghua Tian, Fangfang Zhang, Huijuan Ma, Xingui Chen, Fang Dai, Kai Wang

**Affiliations:** 1 Department of Neurology, First Hospital of Anhui Medical University, Hefei, Anhui Province, PR China; 2 Department of Endocrinology, First Hospital of Anhui Medical University, Hefei, Anhui Province, PR China; VU University Medical Center, NETHERLANDS

## Abstract

**Introduction:**

Cognitive and behavioral impairments are common in patients with abnormal thyroid function; these impairments cause a reduction in their quality of life. The current study investigates the decision making performance in patients with hyperthyroidism to explore the possible mechanism of their cognitive and behavioral impairments.

**Methods:**

Thirty-eight patients with hyperthyroidism and forty healthy control subjects were recruited to perform the Iowa Gambling Task (IGT), which assessed decision making under ambiguous conditions.

**Results:**

Patients with hyperthyroidism had a higher score on the Zung Self-Rating Anxiety Scale (Z-SAS), and exhibited poorer executive function and IGT performance than did healthy control subjects. The patients preferred to choose decks with a high immediate reward, despite a higher future punishment, and were not capable of effectively using feedback information from previous choices. No clinical characteristics were associated with the total net score of the IGT in the current study.

**Conclusions:**

Patients with hyperthyroidism had decision-making impairment under ambiguous conditions. The deficits may result from frontal cortex and limbic system metabolic disorders and dopamine dysfunction.

## Introduction

Hyperthyroidism is a common disease that is clinically diagnosed by a decreased serum thyroid-stimulating hormone (TSH) level and/or increased serum levels of triiodothyronine (T3) and thyroxine (T4); the most common cause of hyperthyroidism is Graves’ disease [1]. The emotion, cognition, and life situations of hyperthyroidism patients have been of concern for clinicians and scholars for a long time [2–8]. A survey study demonstrated that patients with hyperthyroidism had neuropsychiatric impairment, mainly in the aspects of attention, memory, planning, and productivity [9].

In daily life, patients with hyperthyroidism often are nervous, irritable and impulsive. They can not timely recognize the adverse impact of their behavior on their life, work or family, and control their inappropriate behavior and negative emotion. Jablkowska’s [10] study has showed disturbances of executive function, involving impaired prefrontal cortex in patients with hyperthyroidism. Executive function generally refers to a variety of cognitive processes involved in planning, resolving conflicts and controlling behavior [11]. The Stroop Color-Word Test is a simple and frequently used tests to assess the executive control function. Impairment in the Stroop Color-Word Test has been described in patients with attention deficit/hyperactivity disorder (ADHD), patients with anxiety disorder, and patients with alcohol dependence [12–14]. Decision making is also considered to be associated with cognitive processes of executive functions. The processing steps for decision making are supported by specific brain areas and neurotransmitter systems. These brain areas may refer to amygdala, prefrontal cortex, cingulated cortex, and striatum [15–18]. Several studies have showed that the dopaminergic system is critical to decision making process[19, 20]. Animal experiment have also provided some evidence for disturbed reward circuitry function, related to hyperthyroid state [21]. Consequently, patients with hyperthyroidism are prone to making disadvantageous decisions. However, no studies have been published regarding decision making in these patients to date. The principal aim of our study was to bridge the knowledge gap, since decision making is an essential component of the neuropsychological characterization of patients with hyperthyroidism.

Decision making comprises a complex neuropsychological process of weighing and judging short-term and long-term consequence of actions. According to the explicitness of the possibility for reward and punishment, there are two different decision-making situations: decisions with ambiguity and risk conditions [22, 23]. In decision-making under ambiguous condition, the potential outcomes of different options are initially uncertain. The decider has to get some effective information from the feedback of previous trails, and choose the most advantageous alternative among several options in the long run. Impaired performance on decision making with ambiguity has a preference for immediate gains despite negative long-term outcomes. The Iowa Gambling Task (IGT) is frequently used to assess decision making with ambiguity, and appropriately serves as a complementary tool for assessing the executive function domain [19, 24–25]. The IGT has been widely used to evaluate decision making in a variety of psychiatric and neurological diseases.

Hyperthyroidism is caused by the effects of high concentrations of thyroid hormones. Thyroid hormone excess can generate oxidative stress, and long-term exposure to high levels of thyroid hormones damages neuronal cells. Neuroimaging studies have found that patients with hyperthyroidism had reduced grey matter volume using voxel-based morphometry (VBM) [26], and had abnormal cerebral metabolism using localized magnetic resonance spectroscopy (MRS) [27] and positron emission tomography (PET) [28, 29]. Studies on the hypothalamic-pituitary- thyroid (HPT) system have demonstrated that thyroid hormones play a key role in functions of brain. Some researchers focused on a link between thyroid hormones and neurotransmitter systems. The activities of dopamine, noradrenaline and serotonin in brain regions were reported to change in altered thyroid status [30–32]. Dopamine is often associated with many brain functions, such as motivation, desire and addictions [33]. Noradrenaline is known to be important for attention/concentration and vigilance [34], and serotonin has been linked to mood and behaviors including sleeping, feeding and aggression [35]. The complex brain-neurotransmitter imbalance might lead to cognitive and behavioral disorders in patients with hyperthyroidism.

The aims of our study were to measure decision making in patients with hyperthyroidism and to determine the impact of clinical variables on decision making performance. We hypothesized that decision making process would be impaired in patients with hyperthyroidism compared with healthy controls using the IGT, and explored the possible neural mechanisms of this impairment. In addition, we selected patients without anxiety to rule out the interference from that emotional factor.

## Methods

### Participants

All patients with hyperthyroidism (21 women and 17 men, age range: 19–50 y) were recruited from the First Affiliated Hospital of Anhui Medical University. Their educational background ranged from 6 to 16 y; all patients had a Mini-Mental State Examination (MMSE) score > 24. These patients had abnormally high serum T3 (>2.79 nmol/L) and T4 (>140.60 nmol/L) levels and abnormally low TSH (<0.550 μIU/ml) by laboratory examination. Exclusion criteria were as follows: addiction to psychoactive substances, affective disorder (Zung Self-Rating Anxiety Scale (Z-SAS) score >50), other severe neurological and/or psychiatric illness, or head injury revealed by anamnesis.

Forty healthy subjects (21 women and 19 men, ages range: 19–49 y) with normal serum T3, T4, and TSH levels took part in the current study. They were recruited from the public through advertisements, and we paid a fee for their participation. The age, gender, and education of the healthy subjects were matched to the patients with hyperthyroidism. None of the controls had a history of neurologic or psychiatric disorders or endocrine diseases. All participants were right-handed and had normal language expression and understanding skills. All participants provided written informed consent, and the study was approved by the Anhui Medical University Ethics Committee.

### Serum Measurements

After venipuncture, blood was immediately sent to the Endocrinology Laboratory of the First Affiliated Hospital, Anhui Medical University and samples were assayed using chemiluminescence immunoassay. The normal range for T4 was 58.10–140.60 nmol/L; for T3, the normal range was 0.92–2.79 nmol/L; and for TSH, the normal range was 0.550–4.780 μIU/mL. In the chemiluminescence immunoassay, for T4, the sensitivity was 3.87 nmol/L and the coefficients of variation (CV) were less than 5.6%; for T3, the sensitivity was 0.15 nmol/L and the CV were less than 9.4%; for TSH, the sensitivity was 0.001 μIU/mL and the CV were less than 6.6%.

### Neuropsychological Background Tests

For each participant, the Mini-Mental State Examination (MMSE) was used to assess global cognitive functions. The digit span test (forward and backward) was used to assess attention and working memory [36].

The Zung Self-Rating Anxiety Scale (Z-SAS) was a self-report assessment instrument to measure anxiety levels, based on scoring in four groups of manifestations: cognitive, autonomic, motor and central nervous system symptoms. It contained 20 items, and each item was scored on a scale of 1–4 (these replies included “a little of the time”, “some of the time”, “good part of the time”, and “most of the time”). The participants were required to mark how much each item applied to him or her. A total score of less than 50 was considered to be normal.

The Stroop Color-Word Test was used to measure executive function. The participants were asked to read aloud the color (green, red, yellow, blue) of the dots as quickly as they can on page 1. They then read the color of commonly used Chinese characters on page 2 and the reaction time was recorded. Finally, the stimuli consisted of four color names printed in an incongruent color (e.g., the character 红, meaning red color was printed in green) on page 3, and the participants were asked to read the color of the character. The score of Stroop Color-Word Test was calculated by the reaction time of reading page 3 minus the reaction time of reading page 2. A higher score indicated that the participants had worse executive function.

### Iowa Gambling Task (IGT)

The Chinese version of IGT was used to assess decision making under ambiguous conditions. As described in a previous study, the IGT was a computerized version of the gambling task with an automated and computerized method for collecting data. This task required participants to choose one card between four decks of cards labeled A, B, C and D at a time. The behavior of choosing each card from Decks A and B could bring an immediate reward of ¥100, with a maximum possible loss of ¥1250. By choosing Decks C or D, participants only gained ¥50, with a maximum possible lose of ¥250. In general, Decks A and B were disadvantageous because they resulted in a high immediate gain but greater loss over time. Decks C and D were advantageous because smaller losses lead to a positive final balance even if the gains were lower than Decks A and B. Before the task, the participants were not informed of the rules for gains and losses, but they were requested to find them using the feedback after each choice to win as much money as possible. Every participant started the task with ¥2000 shown on the screen and the task had 100 trials. The 100 trials were equally divided into five blocks of 20 trials each. The overall net score (the number of advantageous choices minus the number of disadvantageous choices) and the net score of each block was calculated to investigate IGT performance and decision-making changes during the task.

### Statistical Analysis

Statistical analyses were performed using Statistical Product and Service Solutions (SPSS, version 13.0) (SPSS Inc., Chicago, IL, USA). All data were examined for normality with the Kolmogorov-Smirnov test and for homogeneity of variance with the Levene’s test. The statistical significance of the differences (with the exception of gender) between the patients with hyperthyroidism and healthy controls was compared using an independent samples t-test. The statistical significance of the differences in gender was detected using a chi-square test. Performance differences on the IGT were assessed using a repeated measures analysis of variance (ANOVA) with the block as the within-subject factor and the group (hyperthyroidism group and healthy control group) as the between-subject factor. Correlation analysis between the performance of IGT and the score of the Stroop Color-Word Test and clinical data were examined using Pearson and Spearman’s correlation analysis. Covariance analysis analyzed the effect of Z-SAS score on IGT performance. For all tests, the level of significance was *p* < 0.05.

## Results

Results of the demographic, clinical and neuropsychological test battery are summarized in Table 1. No significant differences in age, gender, education, MMSE, or digit span test (forward and backward) was observed between the hyperthyroidism group and the healthy control group. The hyperthyroidism group patients had higher scores on the SAS and performed significantly worse than healthy subjects on the Stroop Color-Word Test.

**Table 1:** Demographic and background data of the hyperthyroidism group and healthy control group.

	Hyperthyroidism Group (*N* = 38)	Healthy Control Group (*N* = 40)	*t* or χ^2^	*p*
**Age (years)**	30.47 ± 1.00	30.60 ± 8.73	-0.060	0.953
**Gender (F/M)**	21/17	21/19	0.060	0.807
**Education (years)**	10.68 ± 3.21	11.98 ± 3.17	-1.786	0.078
**Disease duration (months)**	7.58±6.80	-	-	-
**T3**	7.55 ± 3.94	1.87 ± 0.47	8.816	<0.001
**T4**	287.46 ± 89.14	98.87 ± 23.25	12.639	<0.001
**TSH**	0.01 ± 0.02	2.19 ± 0.73	-18.854	<0.001
**MMSE**	28.05 ± 1.70	28.13 ± 1.77	-0.184	0.854
**SAS**	36.76 ± 6.57	32.00 ± 6.09	3.323	0.001
**Digit span test (forward)**	7.26 ± 0.92	7.35 ± 0.89	-0.423	0.674
**Digit span test (backward)**	5.05 ± 1.04	5.53 ± 1.15	-1.897	0.062
**Stroop color word test (seconds)**	13.93 ± 6.68	10.87 ± 3.57	2.502	0.015

**Fig 1:**
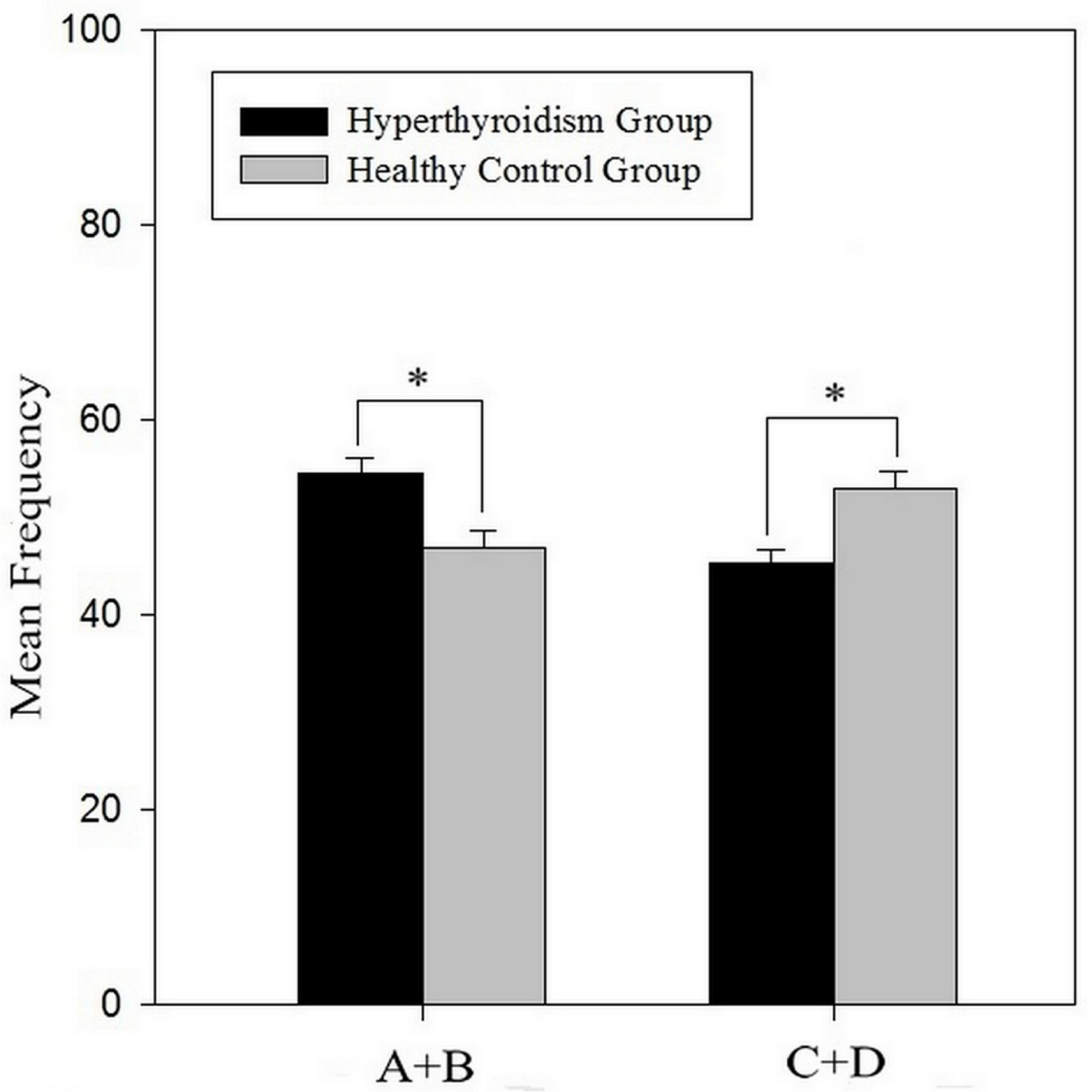
The mean frequency of advantageous choices (A+B) and mean frequency of disadvantageous choices (C+D) for the hyperthyroidism group and the healthy control group. Error bars indicate standard errors of the mean. Items marked with *: *p* < 0.01.

**Fig 2:**
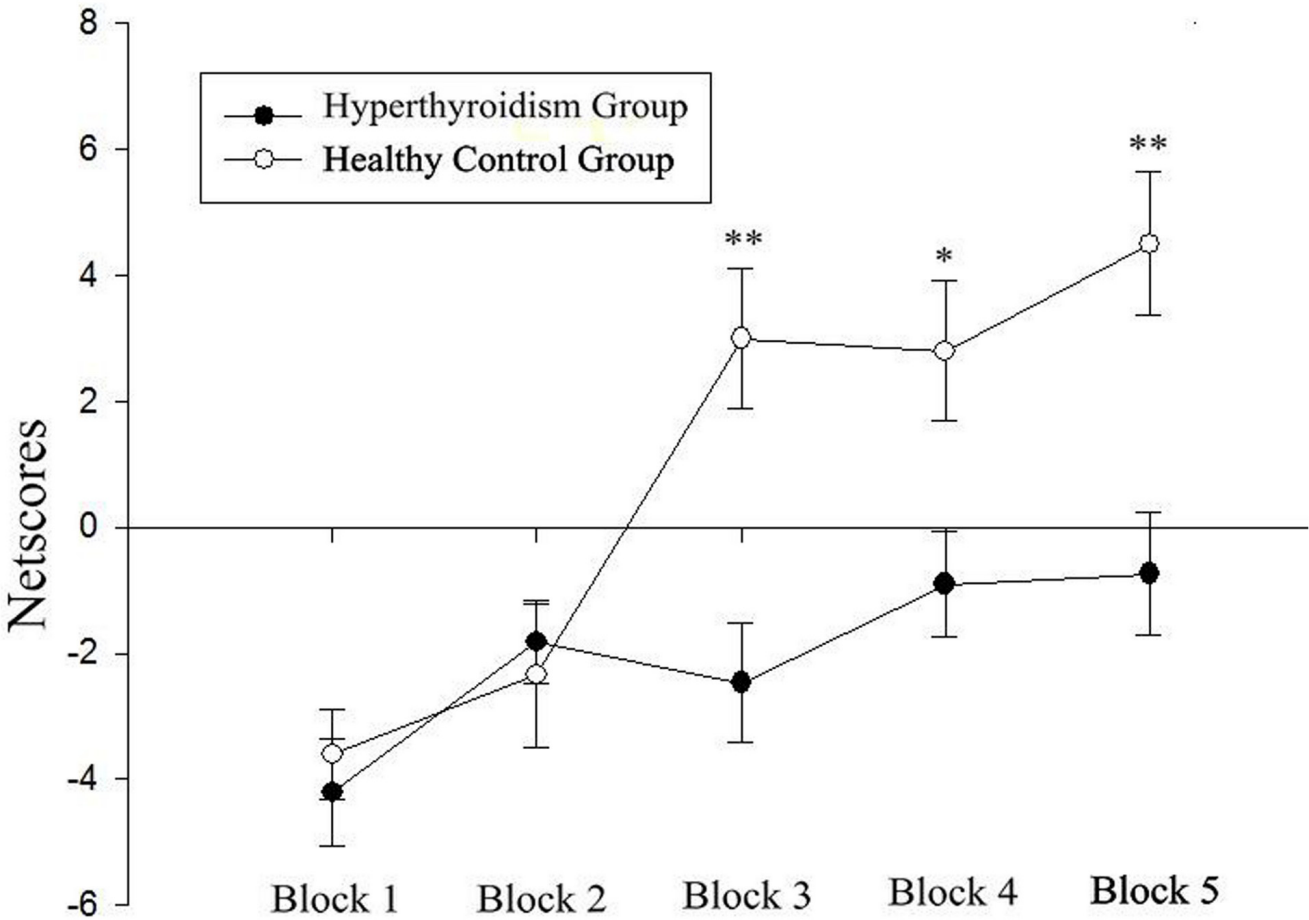
The mean net scores of the 5 blocks, consisting of 20 trials each, for the hyperthyroidism group and the healthy control group. Error bars indicate standard errors of the mean. Items marked with *: *p* < 0.05. Items marked with **: *p* < 0.05.

As shown in Fig 1, a significant difference in the mean frequency of advantageous choices (A+B) and the mean frequency of disadvantageous choices (C+D) was revealed between the two groups. The total net score [(A+B)-(C+D)] of the hyperthyroidism group in the IGT was significantly lower than that of the healthy control group (-9.32 ± 17.97 *vs* 6.20 ± 21.72, *t* (76) = -3.428, *p* = 0.001). The 100 trials were analyzed in blocks of 20 trials. In a repeated-measures ANOVA with the block as the within-subjects factor and the group as the between-subjects factor, there was a significant main effect for block (F (4,304) = 17.214, p < 0.001) as well as for group (F (1, 76) = 10.070, p < 0.002), and there was a significant block × group interaction (F (4,304) = 5.707, p < 0.001). Fig 2 displayed the change curve of the net score, indicating that the hyperthyroidism group and the healthy control group had different decision-making patterns during the IGT. Comparisons of the net score in each block showed significant differences on block 3 (*t* (76) = -3.768, *p* < 0.001), block 4 (*t* (76) = -2.635, *p* = 0.010), and block 5 (*t* (76) = -3.464, *p* = 0.001), while no significant differences were found for block 1 (*t* (76) = -0.550, *p* = 0.584) or block 2 (*t* (76) = 0.405, *p* = 0.687) between the two groups.

Additionally, the total net score of IGT was not correlated with the score of Stroop Color-Word Test (r = 0.050, p = 0.662). No correlations were observed in the hyperthyroidism group between IGT performance and clinical characteristics including disease duration (r = -0.157, p = 0.348) and levels of T3 (r = 0.260, p = 0.115), T4 (r = 0.273, p = 0.097), or TSH (r = 0.072, p = 0.669).

An anxious emotion is important in research focused on cognitive function. Covariance analysis was added to analyze the effect of Z-SAS score on IGT performance. The interaction between groups and the Z-SAS score was not significant (*F* (1,74) = 0.146, *p* = 0.703). The Z-SAS score did not have a significant effect on the disadvantageous choices in the IGT (*F* (1,75) = 0.243, *p* = 0.624).

## Discussion

The main findings of our study were that patients with hyperthyroidism without anxiety had poorer performance on the Stroop Color-Word Test and the IGT, compared with the healthy control group. The patients with hyperthyroidism had a slower reaction time on switching behaviour between congruent and incongruent conditions. The result observed in the Stroop Color-Word Test was consistent with Jablkowska [10] and suggested that patients with hyperthyroidism had executive function impairment. During the decision-making task, We found that patients with hyperthyroidism more frequently opted for decks with a high immediate gain that were defined as disadvantageous choices and paid less attention to higher future punishment than healthy control subjects. They had difficulty finding effective information after choosing the deck randomly, and always had a strong interest in receiving an immediate larger reward and were unable to learn the rule through repeated experience. In the current study, no differences between the two groups were found on the digit span test (forward and backward), which was often used to measure attention and working memory, and the result was in accordance with Vogel’s [4] study who could not find impaired performance on digit span test in the hyperthyroidism group. However, previous studies reported attention and working memory problems in patients with hyperthyroidism using other neuropsychological tests. We could not exclude the possibility that the digit span test was not sensitive enough to reveal the cognitive deficits.

Although our study demonstrated that patients with hyperthydism suffered from executive function and decision making impairments, the concrete mechanism remains unclear. The cognitive impaiments may result from excessive thyroid hormone, which may induce neuronal apoptosis and affect the functionality of certain brain areas [37–39]. The previous study using MRS showed reduced choline/creatine ratio in the frontal lobe of six patients with hyperthyroidism [40]. Fukui [41] speculated that hyperthyroidism might interfere in the integrity of the thyroid-locus ceruleus-frontal lobe system that is useful in maintaining cognitive function. The result of Jablkowska’s [10] research indicated disturbance of executive function in patients with hyperthyroidism associated with impaired prefrontal cortex functionality. Meanwhile, the frontal cortex is involved in decision-making ability as measured by the IGT [42]. Decision making under ambiguous conditions also needs to depend on other brain structures. Early literature have mentioned that the IGT has been related to the limbic loop [23, 43–46]. Schreckenberger [29] and Miao [28] both found that patients suffering from hyperthyroidism showed lower activity in the limbic system using fluorodeoxyglucose positron emission tomography (FDG-PET). Recent research on resting-state functional magnetic resonance imaging (fMRI) demonstrated weaker functional connectivity between the hippocampus and bilateral anterior cingulate cortex (ACC), posterior cingulate cortex (PCC), and medial orbitofrontal cortex (mOFC) in patients with hyperthyroidism [47]. Our study suggested that the altered decision-making strategy in the hyperthyroidism group might be associated with frontal cortex and limbic system dysfunction.

The role of thyroid hormone in many neurotransmitters may conduce to behavioral changes in hyperthyroidism. As previously mentioned, the dopaminergic system is critically involved in decision-making processes and executive function [19]. There was some supporting evidence of a link between dopamine and thyroid state. Mano [48] reported that the level of dopamine decreased significantly in hyperthyroidism. However, as Hassan [31] suggested, hyperthyroidism caused a marked elevation in dopamine level in various brain regions in an animal experiment. Because it is a controversial issue concerning the effect of hyperthyroidism on the level of dopamine in the brain from the previous literature, we speculated that impaired performance of the Stroop Color-Word Test and the IGT in patients with hyperthyroidism might be associated with dopamine dysfunction.

Ruling out interference resulting from anxious emotions, our study excluded patients with anxiety whose score on the Z-SAS was more than 50. However, the hyperthyroidism group still had a higher score on the Z-SAS than the healthy control group, and they failed to learn advantageous strategies in the decision-making task. Impaired decision-making performance under ambiguity in patients with hyperthyroidism seemed to reflect deterioration of both emotional and cognitive processes. They often could not adjust their risky behavior according to potential negative consequences. No correlations were found between clinical features such as disease duration or hormone levels and decision-making performance with the IGT. This might indicate that the changes in the decision-making model were influenced by hyperactive thyroid status and excessive thyroid hormones, not by the patients’ medical history.

## Conclusions

To the best of our knowledge, this study is the first to examine the decision making of adults with hyperthyroidism using the IGT. The results obtained from the current study have shown that the patients with hyperthyroidism had abnormal performance in making decisions under ambiguous conditions. The deficits may result from frontal region and limbic system metabolic disorders and dopamine dysfunction. Our results extend the literature on neuropsychological characterization of patients with hyperthyroidism. In the future, neuroimaging methods and neurobiological techniques will be utilized to explore the association between hyperthyroidism and cognition impairments.

## Supporting Information

S1 Information Background and clinical data of all the patients with hyperthyroidism and healthy control subjects. (XLS)

S2 Information Decision-making performance of 5 blocks, the numbers of choosing advantageous and disadvantageous cards, and total scores in 38 patients with hyperthyroidism and 40 healthy control subjects. (XLS)
